# 

**Published:** 1848

**Authors:** 


### NOTICE TO READERS, CORRESPONDENTS, AND OTHERS.

We have received from Dr. Gregg an original article which will appear in our next number.

We have several other original communications promised.

We have received from S. S. & W. Wood, too late for notice in this number, the following books, which are for sale by Joseph Keene, Chicago, Ill.:

The Young Stethoscopist, or the Student's aid to Auscultation. By Henry J. Bowditch, M.D., one of the Physicians of Mass. Gen. Hospital.

Materia Medica and Therapeutics. By Martyn Paine, A.M., M.D., Professor of the Institutes of Med. and Mat. Med. in the University of New York, &c., &c.

The Obstetrical Remembrancer, or Denman's Aphorisms on Natural and Difficult Parturition, application and use of Instruments, &c. By Michael Ryan, M.D. First American from 9th London edition, with additions by Thomas F. Cock, M.D., visiting physician of New York Lying-in Asylum.

Memoranda on Anatomy, Surgery, and Physiology, forming a Pocket Companion for the Young Surgeon, or for Students preparing for examination. By Mark Noble Bower, Surgeon. Corrected and enlarged by an American Physician.

Opthalmic Memoranda, respecting those diseases of the Eye which are more frequently met with in practice. By John Foote, F. R. C. S. L., &c., &c.

From Lea & Blanchard, a copy of the new edition of Churchill's Midwifery.

Also, 25th Annual Report of the New York Asylum for Lying-in Women.

The Report of the Physicians of the Lunatic Asylum to the Medical Board of Blackwell's Island. Document No. 48, Board of Aldermen, N. Y.

And we have also received our usual exchanges.

We invite the attention of those interested in Medical Colleges, of medical book publishers, surgical instrument makers, &c., to our advertising sheet, which will be extended according to patronage. The terms are, for each page, per annum, $10; one insertion, $5. Half page per annum, $6; one insertion, $3. All advertisements of patent or secret remedies, &c., excluded.

### PROSPECTUS

### OF THE NORTH-WESTERN

### MEDICAL AND SURGICAL JOURNAL.

The rapid extension of the circulation of the Illinois and Indiana Medical and Surgical Journal in ll parts of the great North-West, renders appropriate the change of title above indicated, which made with the commencement of this volume. And while it affords the editors gratifying evidence hat their labors are appreciated, it also encourages them to renewed exertions, hoping that they may, by eriting it, secure the patronage of the profession generally in this extensive region of country.

The first volume will be issued regularly on the first of every other month, beginning with May, multaneously at Chicago and Indianapolis.

It will be neatly printed, with new type, on good paper, each number containing from 88 to 96 pages ctavo, and will at the end of the year form a volume of about 550 pages, with title page and index omplete.

It will be edited by Drs. Herrick, of Chicago, and Evans, of Indianapolis, Professors in Rush Medical ollege, and will be favored by contributions from the other members of the faculty.

The diseases peculiar to the West shall continue to claim especial attention.

Contributions to our pages, from the Physicians of the country are earnestly solicited.

Mr. John W. Duzan, having taken an interest in, and become publisher of the work, will, by his romptitude in business, guarantee against delays in its issue hereafter.

Terms:—Two Dollars in advance, which may be paid to either of the Editors, our authorized Agents, r sent, free of postage, by mail at our risk. Eight dollars, in one remittance, will pay for five new ubscribers.

A list of receipts will be published on the cover of each number, to save the inconvenience of receipting y letter.

Agents---Dr. J. B. Herrick, Vandalia Ill.; Dr. Sample Loftin, and Mr. J. M. Phipps, of Indiana, Ir. Orrin T. Maxson, of W. T.

---

### RECEIPTS FOR THE MEDICAL JOURNAL FOR APRIL, 1848.

| | |
|---|---|
| r. E. Woodruff, Savanna, Ills., | $5 00 |
| r. W. T. Hutchinson White Hall, Ills., | 2 00 |
| r. Dunlap, Delphi, Ind., | 2 00 |
| r. A. E. Ames, Roscoe, Ills, | 2 00 |
| r. J. C. Crawford, Monroe, W. T., | 2 00 |
| r. J. Wasson, New Bedford, Mich., | 2 00 |
| r. Lockwood, Edwardsburgh, Mich., | 2 00 |
| r. Jos. Mansfield, Niles, Mich., | 1 00 |
| r. S. E. Thayer, Galesburg, Mich., | 2 00 |
| Ir. J. Burch, Chicago Ills., | 4 00 |
| r. Brinkerhoff, Chicago, Ills., | 4 00 |
| r. H. L. Terrell, Knightstown, Ia. | 2 00 |
| r. Howland, Ottawa, Ill., | 2 00 |
| r. Coats, Otsego, Michigan, | 2 00 |
| r. Brown, Leoui, do., | 2 00 |
| Dr. P. Maxwell, Chicago, Ills., | 2 00 |
| Dr. Sawyer, Chicago, Ills.. | 4 00 |
| Dr. L. M. Boyce, Chicago, Ills., | 4 00 |
| Dr. E. S. Kumberly, Chiicago, Ills., | 4 00 |
| Dr. John Higgins, Lamoiville Ills., | 2 00 |
| Dr. T. G. Cole, Raymond, W. T., | 1 00 |
| Dr. R. D Manzy, America Ia. | 1 00 |
| Dr. John H. Sanders, Indianapolis, Ia. | 5 00 |
| Dr. J. B. Patterson, Carthage, Ia., | 2 00 |
| Dr. W. J. Larue, Rensellaer, Ia., | 3 00 |
| Dr. W. Brenton, Mount Meridian, Ia., | 2 00 |
| Dr. J. C. Mills, Spring Prairie, W. T., | 2 00 |
| Dr. J. R. Bradway, Delavan, W. T., | 4 00 |
| Dr. Jefferson Miller, Rushville, Indiana, | 2 00 |

